# Priorities in the Protection of Citizens Who Have Fallen into Enemy Hands

**DOI:** 10.1007/s11673-024-10398-0

**Published:** 2024-11-01

**Authors:** Asa Kasher

**Affiliations:** Department of Philosophy, Tel Aviv University, Tel Aviv, Israel

**Keywords:** State priorities; Releasing hostages; Israel; Gaza

The State of Israel was attacked on October 7, 2023, by the Hamas terrorist organization of Gaza. In the attack, about 1150 Israelis and foreign workers were killed and approximately 250 were abducted to Gaza, some of whom were murdered or died while being held captive. More than a hundred were returned as part of an internationally brokered exchange deal. However, 134 of them, including infants, elderly, and sick individuals, are still being held hostage by Hamas or are under its responsibility in Gaza. The Red Cross has not been given access to the hostages; medications were delivered for those in need, but there is no clear indication that they reached all their intended recipients.

Israel responded with a military operation dubbed Operation Iron Swords, its defined objectives being (a) the destruction of Hamas’s military capabilities and the nullification of its governing capabilities, and (b) the return of all the hostages home alive. The operation is currently in full swing, and while objective (a) has been partially achieved in most areas of the Gaza Strip, objective (b) has not been achieved at all. Only three Israelis were rescued in special operations, and three managed to escape on their own but were accidentally killed by IDF gunfire.

Concerns have been raised among the Israeli public about whether the dual objectives of the operation aligned or were contradictory. These concerns have sparked a debate regarding the prioritization of Israel’s efforts: should the release of all the hostages be the foremost priority, or should this objective be deferred until after Hamas’s military and governance capabilities have been successfully neutralized? This article strongly supports the stance that the release of all the hostages should be given supreme, practical, and immediate priority.

The natural inclination to focus on rescuing the hostages has been met with opposition from various directions. First, the argument has been made that prioritizing the rescue of hostages, even at the expense of efforts to dismantle Hamas’s military and governance capabilities, places “the people” and the state at risk. Secondly, there is a claim that releasing Palestinian prisoners in exchange for hostages as part of an international deal poses a real threat because the released prisoners are bound to be dangerous. The first part of this article will demonstrate why we think these claims should not be accepted, while the second part will address additional arguments and explain why we cannot accept them too.

We will first discuss the risks citizens face, presently or under conditions obtained because of an agreement between Israel and Hamas. Then we will discuss dangers, present and forthcoming, on the level of the State of Israel rather than its citizens. The required considerations are different from each other.

A properly functioning democratic state prioritizes the protection of human lives based on the severity, immediacy, and extent to which the threat is being addressed by the bodies tasked with shielding citizens from risk and harm. Undoubtedly, the danger facing the hostages is the most severe: their lives are at risk, the threat is immediate, and it is unaddressed by the state as the latter cannot intervene directly to protect the hostages from the terrorists.

The danger facing an Israeli citizen living in Israel, Jewish or otherwise, due to a ceasefire or even the end of the war, is much lower than the danger facing a Hamas hostage. Under the current circumstances, Hamas cannot launch an attack like that of early October 2023 due to the significant depletion of its capabilities and the protective measures taken by the IDF, the Shin Bet (Israel’s FBI), and the police. The threat of rocket fire persists, but the state is equipped and prepared to protect its citizens from it. Hence, the threat to a citizen, whether a member of “the people” or “just” a local, is negligible compared to the acute and immediate risk facing the hostages.

The danger to citizens due to the release of Palestinian prisoners from Israeli jails depends on the number of those released, the level of threat each of them poses individually, and their dispersion across the various regions after their release. The familiar formula of “all in return for all” is not appropriate under the current circumstances. It was devised in the context of major wars, such as World War II, and is fitting for scenarios where those who are released no longer pose a threat because the war has ended, and no further hostilities are expected. Israel is expected to release prisoners, several for each hostage, based on a list approved by the Shin Bet that considers the threat posed by the prisoners individually and collectively, as well as their dispersion. Israeli citizens always face the threat of terrorist attacks, and this threat is managed by the Shin Bet, the IDF, and the Border Police. In light of this, the list of prisoners to be released must ensure that the existing level of threat, already addressed by state organizations, is maintained. Nevertheless, this threat is immeasurably lower than the extreme and immediate danger faced by the hostages.

In the negotiations for the release of hostages in exchange for prisoners, there could be a significant gap between the number of prisoners Israel is willing to release based on the security requirements stated above, and the number of prisoners Hamas wants and even demands to be released as a condition for the deal. Such a gap could also arise regarding the identity and type of the released prisoners. We will return to these gaps later on.

So far, we have dealt with the dangers facing the state’s citizens due to a short-term or prolonged ceasefire or due to the release of prisoners. Now let us delve into the arguments concerning the dangers facing the state.

The first argument of this type that should be addressed concerns the threat to the state’s very existence. An existential threat is undeniably serious and requires preemptive consideration. However, the connection between the current circumstances and such a threat is tenuous. Even if a ceasefire would allow Hamas to regain its status as a terrorist military organization (as opposed to a guerrilla organization), the likelihood of its success is unrealistic due to the IDF’s effective countermeasures. And even if Hamas did achieve some level of success, this still would not constitute an existential threat. Furthermore, a reasonable deal securing the return of the hostages in exchange for the moderate release of prisoners would not adversely affect Israel’s military activity, and certainly would not create an existential threat.

A related argument highlights the risk of how a ceasefire, particularly an extended one, could influence the attitudes, intentions, and strategies of hostile entities such as Hezbollah in Lebanon or Iran and its proxies in the Middle East. Undoubtedly, the importance of deterrence is crucial whenever a state and its citizens face potential aggression from external entities seeking to do them harm. However, the ethical approach toward deterrence demands acknowledging the fact that the danger involved is not immediate and that care must be taken not to turn some citizens in the present into protective devices for others in the future. Therefore, deterrence should be the by-product of other activities, typically defensive ones, and achieved without casualties incurred from its direct pursuit. Political discourse sometimes portrays a military operation as meant to establish deterrence, but strictly speaking such portrayals are always wrong. Deterrence is a by-product of self-defence. Clearly, a democratic state must not abandon its abducted citizens for the sake of establishing deterrence against future hostilities.

Hamas’s attack on Israel in October 2023 did indeed diminish Israel’s deterrence capability against hostile entities in the Middle East, and Operation Iron Swords is expected to significantly rebuild this deterrence. However, the willingness to take decisive, practical action to rescue citizens abducted by an enemy, through an international deal or alternative methods, does not undermine deterrence. On the contrary, it demonstrates a resilience that reflects the state’s societal resources, particularly in response to overt harm inflicted upon its citizens.

Another similar argument emphasizes Israel’s duty to maintain its independence and sovereignty over its territory. During the October 2023 assault by Hamas, the terrorist organization briefly seized control of a strip of land in southwestern Israel that included civilian settlements and military bases, thereby violating Israel’s territorial sovereignty. Sovereignty is indeed not confined to territorial sovereignty but includes also the control over freedom and safety of citizens, including their staying within the territory of the state.

It is crucial to recognize that pursuing an international deal to rescue the hostages does not imply retroactive acceptance of these two kinds of violation. Rather, it signifies an acknowledgment of the breach while striving to minimize its impact. Operation Iron Swords including the safe return of all the hostages effectively counters the breach of territorial sovereignty, whereas accepting the ongoing captivity of hostages by Hamas amounts to accepting the primary outcome of the violation. Hence, efforts to return the hostages home alive reinforce the state’s sovereignty.

We turn now to arguments that have been used during some public debates that we would like to reject.

First, an argument that prioritizes Israel’s duty to act against Hamas in a manner that restores the “national honour” that was damaged by the October 2023 attack. This argument may partially speak to the erosion of deterrence and the pressing need to strengthen and perhaps even restore it. However, the concern for “national honour” likely carries additional implications, suggesting that states and nations are perceived as entities with emotional capacities and relationships that are driven by the need for emotional expression. I believe that the moral ethos of a democratic state—its values, principles, institutions, and procedures—should not permit emotional motivations to guide its policies or affect its strategies for protecting the lives of its citizens. No reasonable and ethical person wishing to see their democratic state operating effectively would endorse sacrificing the lives of hostages on the altar of “national honour.”

Indeed, emotions can drive individuals and even countries to drastic actions. Occasionally, Israeli citizens express, for example, a desire for “revenge” against Hamas for the crimes it committed in the attack and the acts of murder, rape, and kidnapping it has perpetrated since early October. The desire for revenge in response to injustice is a well-known, widespread, and age-old sentiment. However, the evolution of human morality has seen a shift from the pursuit of revenge to the pursuit of justice, at least in the spheres of domestic arrangements. This approach should not only guide interactions between individuals but also the dynamics between groups. Consequently, the drive for revenge must be restrained, and it certainly cannot justify forsaking the responsibility to protect the lives of the state’s citizens.

Though emotions sometimes are involved in public affairs, on both individual and national levels, they should not take part in national decision-making procedures which must be justifiable on firm grounds of principles and arguments, never on prevalent emotions that are never clear, stable, and justifiable.

The discussion of the various arguments presented above consistently leads us to the conclusion that moral priority must be given to the effort to return the hostages home alive. This can be accomplished through military action and negotiation. Israeli military doctrine stipulates that a “military option” takes precedence over negotiation. The justification for this preference is twofold. First, using military force serves as a deterrent against future abductions by signalling that any efforts to secure advantages through such means will be met with a strong retaliatory action, at the necessary level of violence. Secondly, the use of military means reflects a refusal to tolerate abductions, driving the desire to forcefully overturn their outcome and prevent future abductions.

It is important to emphasize that a military option is not always viable. Taking military action to rescue hostages aligns with the values of the armed forces and the democratic state only if there is a very high likelihood that the hostages will be rescued and a high likelihood that there will be no casualties among the soldiers. If there is no way to guarantee such high odds, it is appropriate to resort to negotiation.

Furthermore, it is important to note that negotiations do not rule out the possibility of identifying and executing a military option at the same time. However, the potential impact of an unsuccessful military action on the viability of continuing the negotiations must be considered carefully.

Having said all this, sometimes the primary strategy involves international negotiations aimed at reaching an agreement that includes the safe return of hostages to their homes as a central part of the deal. This approach is particularly relevant in the current situation, where Hamas and possibly other actors are holding numerous hostages.

Let us examine some ethical aspects of such negotiations.

First, it should be noted that making a responsible ethical assessment of the negotiations is often impossible while they are in progress. The public sphere becomes flooded with claims made by the parties and others designed to influence the proceedings and not necessarily to reveal the truth regarding the current situation and intentions. The truth can be partially gleaned from the outcomes of the negotiation, and largely in memoirs, as far as they can be trusted.

Secondly, every negotiation is conducted within certain parameters. The simplest business negotiation, for example, operates within the parameters of the quality of the goods and their price. Similarly, hostage rescue negotiations are conducted within the parameters of the number of hostages to be returned and the timeline for their release, and on the other hand, the number of prisoners to be released, the crimes for which they were convicted, and critically—the potential risk they pose to the state’s citizens should they return to terrorist activities.

Initially, negotiations can seem deadlocked because neither party is able or willing to meet the demands of the other. In scenarios centred on the rescue of abducted citizens, there is a moral obligation to break through this deadlock. The key strategy to achieve such a breakthrough involves broadening the scope of parameters under discussion. This allows the parties to offer more to gain more, based on their respective interests. In the current context, the field of humanitarian actions could provide additional parameters, as needed.

Thirdly, in conducting judicious and responsible hostage rescue negotiations, the various parameters must be evaluated accurately. Creating needless barriers that hinder reaching an agreement and securing the hostages’ release is unreasonable. For example, in the public discourse about releasing Palestinian prisoners from Israeli jails, some were described as having “blood on their hands,” meaning that they were given life sentences for murders related to terrorist acts. Indeed, these prisoners deserve severe punishment. However, in the context of negotiations, the essential consideration is whether releasing them would pose a real threat to Israel’s citizens. A vast majority of the prisoners released in return to Gil’ad Shalit’s return from Hamas captivity (October 2011) were criminals and not terrorists. In addition, there may not always be a correlation between prisoners having “blood on their hands” and the threat they pose to the state’s citizens in light of their expected involvement in terrorist organizations, given their present conditions and the effects of long periods of serving time in jail.

This leads us to the fourth moral dimension I seek to address, which is the classification of individuals on both sides of the agreement. In the deal executed in November to December 2023, priority was given to the release of children and their mothers. Prioritizing the release of children over adults, particularly when everyone is healthy, is easy to justify based on the understanding that children are more vulnerable and therefore face greater risks to their well-being. In the context of the current war, women were justifiably prioritized over men, largely due to the concern over sexual violence, which is significantly more common against women, though it also occurred against men, though to a lesser degree. The moral guideline for categorizing hostages for release necessitates a logical and fair rationale for any preference, rather than surrendering to external pressures or popular sentiments. Under the given circumstances, prioritizing women has been justified.

The final ethical aspect I wish to briefly address pertains to the means the parties may legitimately employ to advance the negotiations. Negotiation strategies encompass a variety of tactics aimed at furthering interests, some of which are ethically permissible, others of which are not. One such permissible tactic could involve releasing a significant number of prisoners in a way that does not notably affect the level of risk they pose to the state’s citizens. This would go beyond the exchange formula of hostages for prisoners to facilitate gains such as enabling Red Cross visits to each hostage.

A complex idea worth discussing is the public outcry and demonstrations aimed at keeping the necessary level of attention on the urgency of returning the hostages home as soon as possible. At times, this activity meets with criticism for supposedly “raising the price” by intensifying social pressure on decision-makers, potentially weakening their resilience against the enemy. I believe this criticism is generally unfounded. The core framework of the agreement the negotiations aim to achieve is not influenced by minor fluctuations in the pressure exerted on decision-makers. There might be a scenario where public pressure influences decision-makers in situations where a single numerical parameter is being debated, and it is hard to insist on one value when public pressure demands and perhaps even mandates that it should be changed. This is a special situation within the negotiation process that does not apply in the current reality, which involves many and varied parameters. Under the current circumstances, engaging in regular and routine public activity on behalf of the hostages is entirely appropriate.

Among the morally impermissible tactics proposed in the context of trying to advance Israel’s interests, a notable suggestion was to shut down the humanitarian channels benefitting the civilian population in Gaza which is not involved in terrorist activities and does not pose a threat to Israel’s citizens. Providing medicine, water, and food to the general population is not only a legal duty under international law, to which a civilized state ought to be committed, but is also a moral obligation, as human beings and their basic needs are not to be used as instruments to benefit others.

Finally, as self-evident as these points may be, they still need to be stated. A democratic state must act properly and appear to be always acting properly. In a civilized state, the constant public impression that the Prime Minister, certain ministers, and numerous supporting Knesset members are acting as barriers or are being unduly obstructive to the rescue of hostages—for nationalist reasons or even worse, for partisan or personal interests—is unacceptable. Justice must not only be done but also be manifestly done. The same holds for every basic value and principle of democracy. Absolute priority must be given to the release of hostages over long-term and nebulous goals; this must be maintained, and manifestly so, always.

*--

Prof. Asa Kasher is a Professor Emeritus of Professional Ethics and Philosophy at Tel Aviv University and a senior researcher at the Institute for National Security Studies. He was also a member of the Governmental Shamgar Committee on the Release of Israeli Civilians from Enemy Hands.

## Data Availability

Not applicable; no data included.

